# Reforms and innovations in primary health care in different countries: scoping review

**DOI:** 10.1017/S1463423623000725

**Published:** 2024-04-23

**Authors:** Solmaz Azimzadeh, Saber Azami-Aghdash, Jafar Sadegh Tabrizi, Kamal Gholipour

**Affiliations:** 1 Health Policy, Department of Health Policy & Management, School of Management and Medical Informatics, Tabriz University of Medical Sciences, Tabriz, Iran; 2 Health Policy, Medical Philosophy and History Research Center, Tabriz University of Medical Sciences, Tabriz, Iran; 3 Health Services Management, Tabriz Health Services Management Research Center, Tabriz University of Medical Sciences, Tabriz, Iran

**Keywords:** primary health care, Reforms, innovations, scoping review

## Abstract

**Introduction:**

The World Health Organization (WHO) recommends focusing on primary health care (PHC) as the first strategy of countries to achieve the improvement of the health level of communities and has emphasized it again in 2021. Therefore, we intend to take a different look at the PHC system with reform, innovation, and initiative by using the experiences of leading countries and identify practical and evidence-based solutions to achieve greater health.

**Methods:**

This is a scoping review study that has identified innovations and reforms related to PHC since the beginning of 2000 to the end of 2022. In this study, Scopus, Web Of Science, and PubMed databases have been searched using appropriate keywords. This study is done in six steps using Arkesy and O’Malley framework. In this study, the framework of six building blocks of WHO was used to summarize and report the findings.

**Results:**

By searching in different databases, we identified 39426 studies related to reforms in primary care, and after the screening process, 106 studies were analyzed. Our findings were classified and reported into 9 categories (aims, stewardship/leadership, financing & payment, service delivery, health workforce, information, outcomes, policies/considerations, and limitations).

**Conclusion:**

The necessity and importance of strengthening PHC is obvious to everyone due to its great consequences, which requires a lot of will, effort, and coordination at the macro-level of the country, various organizations, and health teams, as well as the participation of people and society.

## Introduction

The primary objective of a healthcare system is to enhance the health status of individuals and populations, enabling active participation in economic and social activities (Franken and Koolman, 2013). The World Health Organization (WHO) strongly advocates for primary health care (PHC) as the foremost strategy for countries to achieve this goal (Starfield et al., 2005). In the early 21st century, there is a renewed emphasis on PHC to attain health objectives, improve health indicators, and effectively address current and future population needs (Hone et al., 2018). A crucial historical milestone in healthcare services provision was the international community’s decision to adopt the primary healthcare strategy, aiming to introduce justice into the health system (King, 2000).

WHO’s annual reports in 2003 and 2008 reiterated the importance of PHC (Van Lerberghe, 2008; World Health Organization, 2003). The global conference on PHC held in October 2018, marking the 40th anniversary of the Almaty Declaration, aims to celebrate its principles and reaffirm political commitment to making PHC the cornerstone of global health coverage and sustainable development goals (World Health Organization, 2018).

After 45 years, PHC has yielded remarkable results, particularly in rural areas, but recent years have presented challenges, especially in urban areas. Key challenges include an aging population, a shift from infectious diseases to non-communicable diseases, evolving healthcare needs, resource instability, a hospital-oriented approach, use of untrained physicians in managerial roles, urbanization, and increased health needs in suburbs (Sheikhattari and Kamangar, 2010, Macinko et al., 2009, Jenkins-Clarke and Carr-Hill, 2001, Tabrizi et al., 2017). Developing countries face additional issues like poor quality of care, inadequate financial resources, insufficient equipment and training, problems in the referral system, and a tendency to allocate resources to higher service levels (Sturmberg et al., 2012).

While most studies on health service quality improvement have focused on diagnostic and medical processes in secondary and tertiary service levels, PHC is also susceptible to process errors, organizational flaws, communication issues, and staff errors. Recognizing the need for change and reform in existing processes is imperative, as studies confirm the severe complications arising from errors in low-quality PHC (Allen, 2000, Azimzadeh et al., 2023, Gholipour et al., 2016). Therefore, a fresh perspective accompanied by change, reform, innovation, and initiative is crucial for PHC.

In recent years, numerous countries have acknowledged the necessity of designing programs and interventions to enhance and innovate their PHC systems. Notably, the United Kingdom has established Accountable Care Organizations (Shortell et al., 2014), Australia has initiated PHC networks (Booth et al., 2016), South Africa has undertaken the ‘Primary Health Care Reengineering’ project and instituted the District Health System (Kautzky and Tollman, 2008), and Estonia has introduced a family medicine-centered PHC model (De Maeseneer, 2016). Additionally, Bosnia has implemented autonomous health teams (Atun et al., 2007), Canada has launched Family Medicine Groups, incorporating local service, network, and network clinic models (Levesque et al., 2010, Pineault et al., 2014b), Turkey has established a Family Medicine model unit (Hone et al., 2017), Brazil has embraced multi-professional teams in basic health units(de Mello et al., 2017), Spain has initiated a multidisciplinary teams-risk stratification model (Doñate-Martínez, 2017), and Kazakhstan has established HealthCity and disease management programs (Sharman, 2014). These initiatives, along with various projects and programs in other countries, underscore the global recognition of the significance of the PHC system and the collective effort to reform and strengthen it.

In 2007, WHO published a framework known as the ‘WHO building blocks’, focusing on the need to strengthen health systems and providing a common conceptual understanding of a health system for assessment and comparison. The framework comprises six building blocks: financing, health workforce, health information system, medical products and technology, service delivery, and governance. This framework serves as a comprehensive tool for assessing health systems, emphasizing the interconnected nature of its components and their collective effectiveness in delivering high-quality, equitable care to all who need it (Alvarez-Rosete et al., 2013, WHO, 2007, Jabeen et al., 2021).

Therefore, leveraging the experiences of leading countries and adopting a fresh perspective on PHC can offer practical, evidence-based solutions to achieve universal health coverage and strengthen the health system for health policymakers.

## Method

This study aims to identify global initiatives and innovations in PHC through a scoping review. It encompasses articles and reports published from January 1, 2000, onward that detail global interventions, initiatives, and best practices for establishing and reinforcing PHC.

In this study, we adopted the Arkesy and O’Malley framework (Arksey and O’Malley, 2005) the first methodological framework for conducting scoping review research, published in 2005. Following this framework, we executed six steps: identification of the research question, identification of related studies, screening and selection of studies, categorization of data, and, finally, summarization and reporting of results, along with the provision of practical tips and advice.

### First step: Identifying the research question

The primary research question is, ‘How are innovations and reforms related to PHC in different countries?’ This encompasses specific queries:

- In which countries are primary healthcare innovations and initiatives prevalent?
- What are the goals/aims of primary healthcare innovations and reforms in different countries?
- For which areas and services have primary healthcare innovations and reforms been applied?
- What mechanisms for financing and payment are considered in primary healthcare innovations and reforms in different countries?
- What are the results and achievements of primary healthcare innovations and reforms in the world?
- What limitations did primary healthcare innovations and reforms face?

### Second step: Identifying related studies

We conducted a search using Scopus, Web Of Science, and PubMed databases from January 1, 2000, to December 31, 2022. Keywords were determined through similar studies, expert opinions, librarians’ insights, and Medical Subject Headings (MeSH). Primary search keywords included the following: primary health care, primary health services, basic health care, public health, primary care, reform, Strength*, Transform*, innovation, initiative, etc. Additionally, a manual search of journals, references of selected articles (Reference of Reference), review of organizational reports, published government documents, and websites was conducted. Inclusion criteria for articles and reports included being published after 2000, published in English, and related to innovation in PHC. In this study, innovation refers to programs, plans, interventions, initiatives, and any new changes aimed at reforming and strengthening the primary healthcare system. The innovative model of PHC encompasses changes and innovations in organizational structure, communication between system components, service packages, human resources, main goals and approaches of care, payment systems and resource management, monitoring and evaluation approaches, as well as management and leadership of the PHC system to enhance performance and adapt to the existing requirements and conditions of the country.

***Search strategy in PubMed***: (“Primary health care“[Title/Abstract] OR “Primary healthcare“[Title/Abstract] OR “Primary care“[Title/Abstract] OR “Primary health service“[Title/Abstract] OR “Public health care“[Title/Abstract] OR “Public healthcare“[Title/Abstract] OR “family medicine“[Title/Abstract] OR “family physician“[Title/Abstract] OR “family practice“[Title/Abstract] OR “Public health service“[Title/Abstract]) AND (“loattrfull text“[Filter] AND 2000/01/01:2022/12/31[Date - Publication] AND “english“[Language]) AND ((“reform“[Title/Abstract] OR “innovat*“[Title/Abstract] OR “transform*“[Title/Abstract] OR “initiat*“[Title/Abstract] OR “strengthen*“[Title/Abstract]) AND (“loattrfull text“[Filter] AND 2000/01/01:2022/12/31[Date - Publication] AND “english“[Language]))

### Third step: selection/screening of studies

All stages of article selection and screening were independently conducted by two members of the research team. In the initial stage, any disputed cases were resolved through discussion, and if necessary, a third person with more information and experience was consulted. The first step involved reviewing the titles of all articles, excluding those not aligning with the study’s objectives. Subsequent steps involved studying the abstracts and full texts to identify and exclude studies that met the exclusion criteria, such as poor relevance to study objectives, insufficient information, and focus on specific groups/diseases/ items.

Considering variations in the structure of providing PHC across countries, two researchers examined the information in each study to determine its relevance to PHC and whether it included innovation. Based on the information presented and agreement between the researchers, decisions were made regarding the selection of articles or reports. Any discrepancies between the two researchers were resolved through discussion. In cases where no agreement was reached, a third person with higher expertise and experience in the field of PHC was consulted.

Endnote X9 resource management software was utilized for organizing, reading titles and abstracts, and identifying duplicates. The 2020 PRISMA flowchart was employed to report the results of the selection and screening process.

### The fourth step: segmentation of data

Following the elimination of articles that did not meet the inclusion criteria, the full text of all qualifying articles underwent a comprehensive review. The research team designed a data extraction form to gather essential information, including interventions, strategies, achievements, outcomes, innovation components, etc.

Initially, the data extraction form was manually created in the software environment of Microsoft Word 2010. To refine the form, data from three articles were extracted as a test, and any deficiencies or issues were addressed. Two individuals independently extracted the information, and any ambiguities were resolved through consultation with members of the research team. Extracted information encompassed details such as the name and year of the innovation/reform, country, the aim of the innovation/reform, target group, management, financing and payment, type of services, service provider staff, results of the innovation/reform, policy /considerations, and limitations.

In cases where discrepancies arose between the two individuals, consensus was reached through discussion. If no agreement was reached, the contested cases were referred to a third person with greater expertise and experience in the field.

Subsequently, the findings were summarized and classified based on the WHO’s framework of six building blocks, illustrating the changes and performance of health systems. Notably, our analysis revealed no reportable findings for the item of medical products.

### The fifth step: Summarizing and reporting the results

Following the extraction of information using the data extraction form, a manual analysis was conducted, and the findings were summarized and reported using the content analysis method. Thematic analysis, a valuable method for qualitative data analysis, was employed to identify, analyze, and report patterns (themes) in the text (Graneheim and Lundman, 2004). Data coding was carried out independently by two researchers. Following the extraction of information using the data extraction form, a manual analysis was conducted, and the findings were summarized and reported using the content analysis method. Thematic analysis, a valuable method for qualitative data analysis, was employed to identify, analyze, and report patterns (themes) in the text. Data coding was carried out independently by two researchers.

The stages of data analysis and coding were as follows: becoming familiar with the text of the articles (immersion in the results of the articles), identifying and extracting the primary fields (articles mostly related to the primary fields), categorizing the articles within specified fields, reviewing and enhancing the results of each field using the findings of the articles, and ensuring the reliability of the fields and extracted results in each field. In cases of disagreement between the two researchers, resolution was reached through discussion, and disputed issues were addressed between the two coders. In the absence of agreement, the dispute was referred to a third person.

The findings were subsequently summarized and classified based on the WHO’s framework of six building blocks (WHO, 2007), illustrating the changes and performance of health systems. The six building blocks, constituting a health system, encompass service delivery, health workforce (human resources), information (data and data systems), medical products, vaccines and technologies, financing, and leadership and governance (stewardship). Strengthening these six building blocks is essential to achieving the overall goals of a health system, including improved health, responsiveness, social and financial risk protection, and improved efficiency. Intermediate goals such as access, coverage, quality, and safety were considered as ‘aims’, while overall goals were referred to as ‘outcomes’. Additionally, two categories, ‘policy /considerations’ and ‘limitations’, were incorporated into the analysis and report. According to our findings, no reportable information was obtained for the item of medical products.

### The sixth step: Providing practical guidance and recommendations

Based on the extracted results and the opinions of the research team members, guidance and recommendations were formulated in the form of an article discussion.

## Results

**Figure 1.**
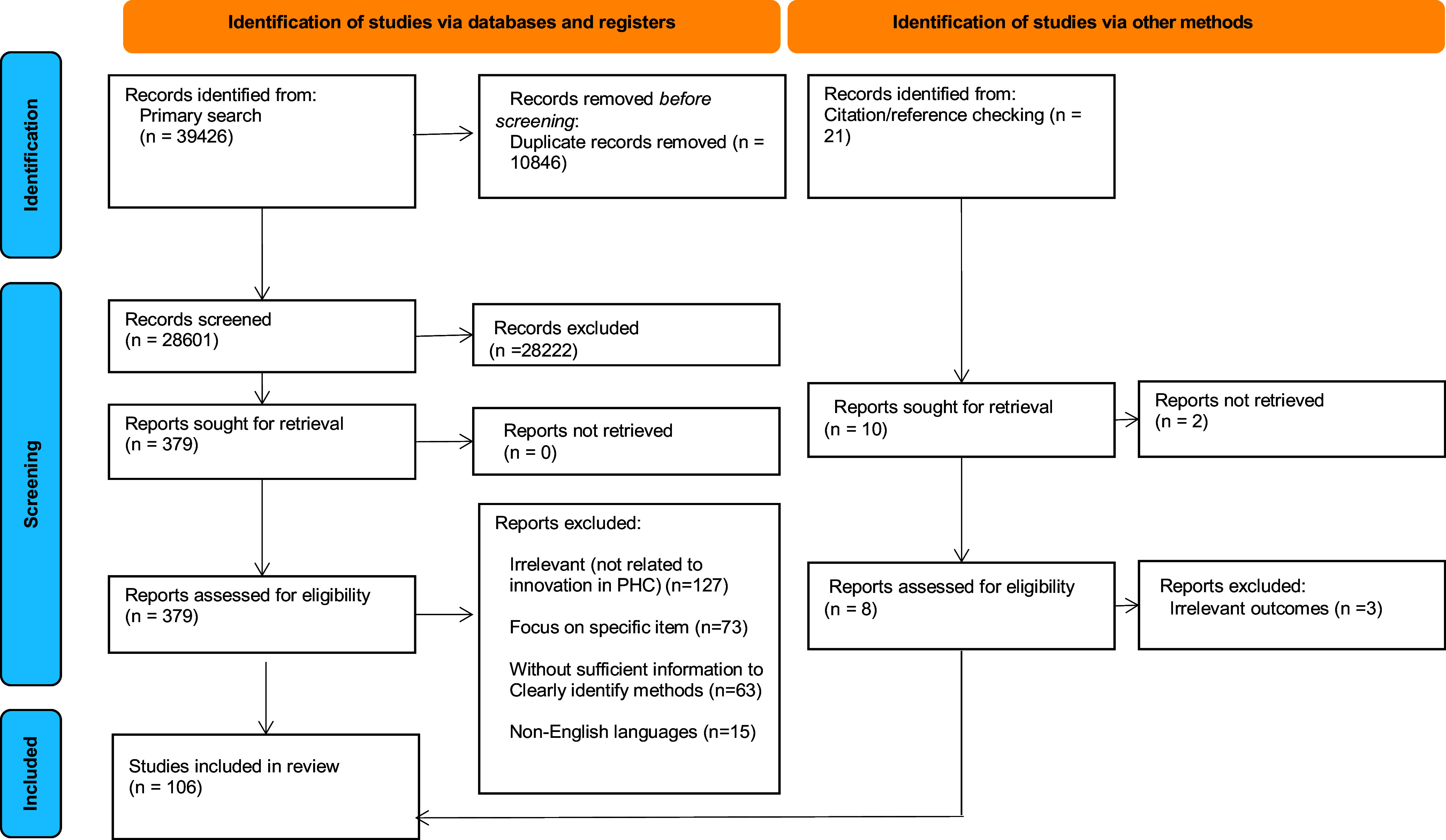
Diagram of a scoping review.

Through database searches, we initially identified 39,426 articles, and post-duplicate removal, 28,601 articles remained. Subsequently, these records underwent screening based on title, followed by scrutiny of abstracts and full texts. Upon completing this screening phase, 106 studies were included in the analysis. The search and selection process are visually presented in Figure 1.

The necessary data were then extracted from these final 106 studies, utilizing a data extraction table, which led to the identification of 55 innovations/reforms. Subsequently, data analysis was conducted from various dimensions.

Our study’s findings reveal that the most significant number of reforms and innovations within the PHC systems of different countries occurred during the period 2000–2008. Canada emerged as the leader in implementing reforms, boasting 10 significant reforms, followed by Estonia with six reforms.

**Figure 2.**
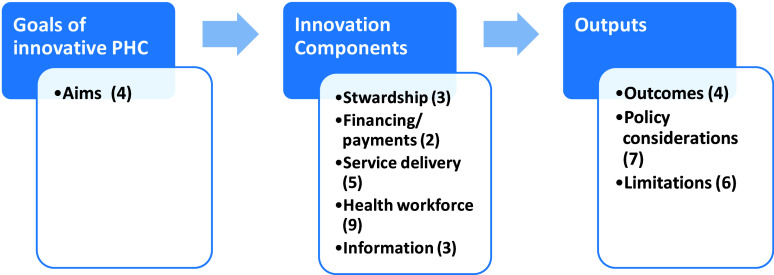
Classification of study findings and the respective number of main subcategories for each subject.

Qualitative analysis categorized the study’s findings, as depicted in Figure 2, and these categories will be expounded upon in detail.

In the analysis of innovation/reform ‘Aims’, using the content analysis method, 63 codes were extracted which were classified into 4 categories (Access, Coverage, Quality, and Safety) with 32 subcategories based on six building block framework (Table 1). Improving the quality of care, improving the effectiveness of monitoring and evaluations, improving financial and geographical access, improving the exchange of information at different levels, early diagnosis of diseases, improving the quality and quantity of follow-ups, etc. are among the most frequent aims mentioned in the studies for PHC.

**Table 1.** Aims of primary healthcare initiatives

Aims	Instances/Examples in studies	References
**Quality**	1. Facilitate innovation in primary care	(Abos Mendizabal et al., 2013)
	2. Create an integrated model to improve the quality of PHC	(Garralda et al., 2016, Llobera et al., 2018)
	3. Improve the quality of care;	(Levesque et al., 2010, Pineault et al., 2014a, Breton et al., 2011, Gilbert et al., 2015)
	4. Improve the quality of primary care by focusing on chronic disease management	(Beaulac et al., 2017)
	5. Create new approaches to monitoring and evaluation	(Ahmedov, 2014, Nickell et al., 2020)
	6. Focus on efficiency, equity & accountability	(Atun et al., 2016, Biscaia and Heleno, 2017, Atun et al., 2006)
	7. Create a new care model for the coordination of cares	(Nickell et al., 2020)
	8. Focus on incentives & payments	(Tatar et al., 2011)
	9. Improving participation & satisfying both professionals and users.	(Biscaia and Heleno, 2017)
	10. Use evidence & needs to provide services	(Ghiotto et al., 2018)
**Safety**	1. Higher quality health care with transparency and accountability	(Atun et al., 2006)
	2. Improved coordination of care transitions.	(Peikes et al., 2018)
	3. Information exchange in care	(Organization, 2016, Unit, 2019, Atun et al., 2016)
	4. Early identification & diagnosis of patients	(Doñate-Martínez, 2017, Prades and Borràs, 2011)
	5. Improve the use of information	(Beaulac et al., 2017)
	6. Focus on information exchange between providers	(Atun et al., 2016)
**Coverage**	1. Patient management and follow-up	(Breton et al., 2011, Gilbert et al., 2015, Levesque et al., 2010, Pineault et al., 2014a)
	2. Improve care coordination	(Aggarwal and Williams, 2019, Ditta and Ahmed, 2019, Nickell et al., 2020, Peikes et al., 2018, NHS Providers, 2018, Syed et al., 2020)
	3. Assessment and management of patients	(Atun et al., 2016, De Coster et al., 2010, Gilbert et al., 2015, Levesque et al., 2010)
	4. Improve the referral system	(Badora-Musiał et al., 2021)
	5. Focus on organization & process	(Carvalho et al., 2014, Mendes et al., 2018)
	6. Expand primary healthcare	(Fausto et al., 2018)
	7. Prioritize services	(Booth et al., 2016, Campbell et al., 2011, Lester and Campbell, 2010)
	8. Focus on primary care at the community level	(Bheekie and Bradley, 2016, Lewis and Chana, 2018)
	9. Decreased hospital care	(NHS Providers, 2018)
	10. Use of non-physician health staff	(Cevik and Kilic, 2018)
	11. Use evidence & needs to provide services	(Ghiotto et al., 2018, Hailemariam et al., 2018)
**Access**	1. Improves access to primary care	(Breton et al., 2011, Gilbert et al., 2015, Levesque et al., 2010, Pineault et al., 2014a)
	2. Geographical access	(Aggarwal, 2009, Aggarwal and Williams, 2019, Atun et al., 2006, Beaulac et al., 2017, Glazier, 2012, Goldman et al., 2010, Kantarevic et al., 2011, Levesque et al., 2010, Pineault et al., 2014a)
	3. Financial access	(Aggarwal and Williams, 2019, Atun et al., 2016, Atun et al., 2006, Beaulac et al., 2017, Goldman et al., 2010, Lahariya, 2019, Bhattacharyya et al., 2011, Kanavos et al., 2009)
	4. Providing comprehensive care	(Aggarwal and Williams, 2019, Atun et al., 2016)
	5. Upgrading health facilities	(Krishnan and Nair, 2021)

In the analysis of the ‘Stewardship/Leadership’ component, encompassing countries that have undergone changes and reforms in this domain, 33 codes were identified and subsequently categorized into three overarching groups: ‘Level of Management’, ‘Mechanism’, and ‘Interaction (with gov)’. Each of these three categories is further divided into subcategories that delineate various levels and types of stewardship in PHC, the mechanisms and approaches for managing these responsibilities, and the ways in which these organizations interact with governments. Each category comprises several components, providing a comprehensive overview, as outlined in Table 2.

**Table 2.** Stewardship of PHC initiatives

Stewardship/leadership structure	Classifications of stewardship/leadership	Instances/examples in studies	References
**Level of management**	1. Governmental	Government – Ministry of Health	(Aggarwal and Williams, 2019, Ahmedov, 2014, Atun et al., 2016, Lahariya, 2020, Lin et al., 2015)
	2. Regional–Governmental	Establish a federal ministry and decentralize services, District management teams (DMTs)	(Atun et al., 2007, Atun et al., 2006, Ouimet et al., 2015, Pineault et al., 2014a, de Mello et al., 2017, Rabkin et al., 2015)
	3. Public-Private	Governance by community-based, provider-based, or a mix of both, Contracts between health managers of employees FP	(Aggarwal and Williams, 2019, Goldman et al., 2010, Badora-Musiał et al., 2021, Sagan et al., 2022)
	4. Private	Under the management and guidance of physicians, Medical Corporation managed by providers	(Ditta and Ahmed, 2019, Syed et al., 2020, Tatar et al., 2011, Vats et al., 2013)
	5. Municipal	Municipal planning	(Bueno et al., 2013, Lahariya, 2019, Nickell et al., 2020)
**Mechanism**	1. Collaborative	Ministry of Health and the NHF (National Health Fund), Minister of Health, and other Senior Government officials	(Aggarwal and Williams, 2019, Badora-Musiał et al., 2021, Booth et al., 2016, Carvalho et al., 2014, Cevik and Kilic, 2018, Goldman et al., 2010, Lahariya, 2019, Lin et al., 2015, Mendes et al., 2018, Unit, 2019)
	2. Delegated	Under the administration of physicians	(Aggarwal, 2009, Aggarwal and Williams, 2019, Atun et al., 2006, Bheekie and Bradley, 2016, Bueno et al., 2013, Ditta and Ahmed, 2019, Glazier, 2012, Lahariya, 2019, Lewis and Chana, 2018, Nickell et al., 2020, Syed et al., 2020, Tatar et al., 2011, Vats et al., 2013, Levesque et al., 2010, Pineault et al., 2014a)
**Interaction (with gov)**	1. Coordination	Coordination by government	(Aggarwal, 2009, Aggarwal and Williams, 2019, Glazier, 2012, Goldman et al., 2010)
	2. Participatory	It was initiated by the Montréal Regional Health Agency as a complement to FMGs	(Aggarwal and Williams, 2019, Levesque et al., 2010, Pineault et al., 2014a)
	3. Independent	Independent of government, GP-led Clinical Councils, and Community Advisory Committees, Medical Corporation	(Booth et al., 2016, Bueno et al., 2013, Carvalho et al., 2014, Ditta and Ahmed, 2019, Lahariya, 2019, Lewis and Chana, 2018, Mendes et al., 2018, Nickell et al., 2020, Syed et al., 2020, Tatar et al., 2011, Vats et al., 2013)
	4. Dependent	Under the leadership of the government bodies, Minister of Health and other Senior Government officials	(Atun et al., 2016, Atun et al., 2006, Badora-Musiał et al., 2021, Bheekie and Bradley, 2016, Cevik and Kilic, 2018, Hone et al., 2017, Lahariya, 2019, Ahmedov, 2014, Unit, 2019, Lin et al., 2015, Atun et al., 2007)

In the analysis of the ‘Financing & Payment’ component, examining countries that have initiated changes and reforms in the realm of PHC, we extracted 45 codes. These codes were subsequently organized into 8 overarching themes/categories, with the financing part encompassing ‘State Government’ and Taxation’, and the payment part featuring ‘Salary’, ‘Pay-for-Performance’, ‘Combined Payments’, ‘Capitation’, ‘Budget’, and ‘Bonus’, as illustrated in Table 3.

**Table 3.** Financing & payment of PHC initiatives

Financing	Different kinds of payment/financing	References
**Payment**	1. Capitation	(Atun et al., 2016, Atun et al., 2006, Doñate-Martínez, 2017, Prades and Borràs, 2011, Tatar et al., 2011, Vats et al., 2013, Biscaia and Heleno, 2017, Cevik and Kilic, 2018, Espinosa-González and Normand, 2019, Ghiotto et al., 2018, Matulis and Lloyd, 2018, Ryan et al., 2015)
	2. Budget	(Badora-Musiał et al., 2021, Lewis and Chana, 2018, Matulis and Lloyd, 2018, Ryan et al., 2015, Vats et al., 2013)
	3. Pay-for-performance	(Beaulac et al., 2017, Biscaia and Heleno, 2017, Campbell et al., 2011, de Mello et al., 2017, Lester and Campbell, 2010, Tatar et al., 2011, Unit, 2019)
	4. Salary	(Cevik and Kilic, 2018)
	5. Bonus	(Atun et al., 2016, Atun et al., 2007, Espinosa-González and Normand, 2019)
	6. Combined payments	(Aggarwal and Williams, 2019, Atun et al., 2006, Glazier, 2012, Goldman et al., 2010, Kantarevic et al., 2011, Kezunovic et al., 2013, Levesque et al., 2010, Nickell et al., 2020)
**Financial resource collection**	1. State government	(Pineault et al., 2014a)
	2. Taxation	(Ditta and Ahmed, 2019)

Among the various payment methods identified in our findings, ‘Pay-for-Performance’ emerged as the most prevalent. This method was evident in PHC innovations and reforms in countries such as Canada, Brazil, Hungary, Portugal, England, and Sri Lanka, where payments are typically contingent on the attainment of diverse indicators, including quality indicators. Following closely, ‘Capitation’ and ‘Combined Payments’ were noted as the next frequently employed payment methods, identified in various studies as suitable mechanisms for remunerating PHC services.

In the analysis of the ‘services delivery’ component, in the countries that have made changes and reforms in this field of PHC, 84 codes were extracted which were classified into 5 categories/themes (Promotion, Prevention, Diagnosis, Treatment, and Rehabilitation). Among the types of services added or strengthened in new PHC systems, prevention and promotion services had wider and more diverse programs, and more studies have pointed to them. A number of studies have also spoken about the need to include rehabilitation care in PHC. These contents are detailed in Table 4.

**Table 4.** Service delivery in PHC initiatives

Service delivery	Instances/examples in studies	References
**Promotion**	School vaccinations, environmental health services, surveillance of communicable diseases, cancer screening programs and health promotion activities and obesity, healthy lifestyle, physical exercise behavioral health	(Atun et al., 2016, Bheekie and Bradley, 2016, Cevik and Kilic, 2018, Ditta and Ahmed, 2019, Espinosa-González and Normand, 2019, Kanavos et al., 2009, Syed et al., 2020)
**Prevention**	Screening, health checks, smoking cessation, exercise, weight reduction and diet, family planning, Regular annual checkups, family Health, chronically ill, pregnant women and healthy neonates and immunizations	(Atun et al., 2016, Cevik and Kilic, 2018, Ditta and Ahmed, 2019, Espinosa-González and Normand, 2019, Ghiotto et al., 2018, Kanavos et al., 2009, Lahariya, 2019, Syed et al., 2020)
**Diagnosis**	Radiology laboratory	(Ditta and Ahmed, 2019, Syed et al., 2020)
**Treatment**	Home visit, combination of traditional Chinese and Western medicines chronic disease, dental,	(Cevik and Kilic, 2018, Ditta and Ahmed, 2019, Espinosa-González and Normand, 2019, Lin et al., 2015, Syed et al., 2020)
**Rehabilitation**	Mental health,	(Booth et al., 2016, Unit, 2019)

In the analysis of the ‘workforce’ component, in the countries that have made changes and reforms in this field of PHC, 85 codes were extracted which were classified into 9 categories/themes (physicians, specialist, psychologist, pharmacist, nurse, dentist, community health worker, clinical staff, Administrative staff) as shown in Table 5. The addition of personnel such as nutritionists, psychologists, dentists, and community health worker, to PHC has been an interesting initiative in different countries.

**Table 5.** Health workforce of PHC initiatives

Health workforce	Sub-category	References
**Physician**		(Aggarwal and Williams, 2019, Breton et al., 2011, Doñate-Martínez, 2017, Gilbert et al., 2015, Glazier, 2012, Jaakkimainen et al., 2011, Kantarevic et al., 2011, Kralj and Kantarevic, 2013, Levesque et al., 2010, Prades and Borràs, 2011)
**Nurse**		(Aggarwal and Williams, 2019, Doñate-Martínez, 2017, Garralda et al., 2016, Glazier, 2012, Goldman et al., 2010, Kantarevic et al., 2011, Levesque et al., 2010, Llobera et al., 2018, Nickell et al., 2020, Prades and Borràs, 2011)
**Specialist**	Dieticians, biomedicine, radiologist, specialist for family medicine as a gatekeeper	(Atun et al., 2006, Badora-Musiał et al., 2021, Carvalho et al., 2014, Ditta and Ahmed, 2019, Mendes et al., 2018, Syed et al., 2020)
**Psychologist**		(Carvalho et al., 2014, Mendes et al., 2018)
**Pharmacist**		(Carvalho et al., 2014, Mendes et al., 2018)
**Dentist**		(Booth et al., 2016, Carvalho et al., 2014, Ditta and Ahmed, 2019, Krishnan and Nair, 2021, Mendes et al., 2018, Syed et al., 2020)
**Clinical staff**	Midwives, radiographers, laboratory technicians,	(Cevik and Kilic, 2018, Espinosa-González and Normand, 2019, Unit, 2019)
**Community health worker**		(Bheekie and Bradley, 2016, Rabkin et al., 2015)
**Administrative Staff**		(Ghiotto et al., 2018)

In the analysis of the ‘Information’ component, in the countries that have made changes and reforms in this field of PHC, 14 codes were extracted which were classified into 3 categories/themes (Human resource development, Quality improvement, Resource management) as shown in Table 6. Electronic prescriptions, use of electronic health records, creation of extensive databases, paying attention to the social variables of patients using databases and software were some of the initiatives considered in different countries.

**Table 6.** Information on PHC initiatives

Information	Instances/examples in studies	References
**Human resource development**	1. Innovation via using the software.	(Abos Mendizabal et al., 2013)
	2. Implementing a wide range of care plans for professionals in accordance with patient’s health and social needs using variables available at electronic Health Information Systems	(Doñate-Martínez, 2017, Prades and Borràs, 2011)
	3. Providers’ access to and use of information – increasing awareness of guidelines and enabling monitoring	(Beaulac et al., 2017)
**Quality improvement**	1. Generate extensive leverage of the database	(Abos Mendizabal et al., 2013)
	2. Providers’ access to and use of information – increasing awareness of guidelines and enabling monitoring	(Beaulac et al., 2017)
	3. Nationwide E-health system (allowing information exchange between clinical)	(Atun et al., 2016)
	4. Integrated electronic health records	(Atun et al., 2016)
	5. E-prescriptions	(Atun et al., 2016, Atun et al., 2007)
	6. Digital imaging and laboratory tests	(Atun et al., 2016)
	7. Access to data by research and managerial professionals, with data confidentiality governed by strict protection laws, while providing patients access and control of their records	(Atun et al., 2016, Atun et al., 2007)
	8. Symptom triage, health information, and help with accessing healthcare services.	(De Coster et al., 2010)
	9. Use of electronic health records	(Atun et al., 2016, de Mello et al., 2017, Ditta and Ahmed, 2019, Syed et al., 2020)
**Resource management**	1. Improve efficiency by reducing paperwork and duplication	(Atun et al., 2016)
	2. Develop using cost-effective technology (computer-based medicine record maintenance, software for prescription writing, technology-based tablets for conducting laboratory tests, …)	(Lahariya, 2019, Lahariya, 2020)

In the analysis of the ‘Outcomes’ component, in the studies that have mentioned the result/effect of reforms in PHC, 31 codes were extracted which were classified into 4 categories/themes based on the framework of six building blocks (Improved health, Improved efficiency, Responsiveness, and Social & financial protection) which are detailed in Table 7. Reducing hospitalization, increasing life expectancy, reducing the use of medical services, guideline-oriented care, increasing physician productivity, etc. were some of the outcomes of interest for innovation in PHC.

**Table 7.** Outcomes of PHC initiatives

Outcomes	Instances/examples in studies	References
**Improved health**	1. Improve the quality of care and reduce the hospitalization 2. Improvements in the indicators reflecting child and maternal health 3. Increasing life expectancy 4. Mobilization for better health, 5. Improve health outcomes, through better prevention and follow-up 6. Health promotion	(Badora-Musiał et al., 2021, de Mello et al., 2017, Tatar et al., 2011, Ahmedov, 2014, Atun et al., 2016, Atun et al., 2007, Bheekie and Bradley, 2016, Biscaia and Heleno, 2017, Lavis et al., 2002)
**Improved efficiency**	1. Reduction of utilization of services 2. An alignment between different organizational levels 3. Increases physician productivity – physicians have lower referral rates 4. Optimize payment methods 5. More cost-effective prescribing 6. Improve the efficiency and effectiveness of medical services 7. Administration of separate revenue and expenditure 8. Reduce the use of secondary care 9. Reduce avoidable hospital admissions and elective activity 10. Significant increase in PHC utilization	(Abos Mendizabal et al., 2013, Atun et al., 2016, Atun et al., 2006, Biscaia and Heleno, 2017, Booth et al., 2016, Cevik and Kilic, 2018, Espinosa-González and Normand, 2019, Kanavos et al., 2009, Kantarevic et al., 2011, Levesque et al., 2010, Lewis and Chana, 2018, Lin et al., 2015, Pineault et al., 2014a, Polit et al., 2007, NHS Providers, 2018)
**Responsiveness**	1. Increasing awareness of guidelines and enabling monitoring 2. Create a comprehensive interprofessional team of healthcare professionals 3. E-health 4. Strengthening of research and development 5. Guiding patients through the health system 6. Use evidence to provide services	(Atun et al., 2016, Badora-Musiał et al., 2021, Bheekie and Bradley, 2016, Nickell et al., 2020)
**Social & financial protection**	1. Improving access to physician services. 2. Expansion of the PHC network 3. Create a more holistic system of care.	(Booth et al., 2016, Cevik and Kilic, 2018, de Mello et al., 2017, Kantarevic et al., 2011, Kralj and Kantarevic, 2013)

In the analysis of ‘Policies/consideration’, in the studies that have mentioned political considerations of PHC reform, 36 codes were extracted which were classified into 7 categories/themes (using facilitator, Gradual change, Flexibility& Integration with existing systems, Evidence-based performance, communication, and interaction & team working, Government/ Political support, Empowerment), the details of which are given in Table 8. Focusing on initial changes before making major changes, flexibility in designing structures and teams, participation of service providers and creating a suitable and flexible environment for implementing innovations, were the common policies/considerations used in different countries to reform and innovate in Primary health care.

**Table 8.** Policies/ considerations in PHC initiatives

Policies/consideration	Instances/examples in studies	References
**Using facilitator**	The facilitator role played by a ‘neutral’ agent has been key in facilitating the debate between professionals at every organizational level Formulation of policies to facilitate and guide the managerial activity and training of managers.	(Abos Mendizabal et al., 2013, Carvalho et al., 2014, Mendes et al., 2018)
**Gradual change**	Focus on the initial change before the main change Detailed assessments of the regional population’s health needs during the reform, a market analysis of local healthcare services, and the evaluation of the quality and performance of new services Renovation of the previous system by: • Strengthening follow-up services • Strengthening the national health information system • Strengthening the surveillance system	(Booth et al., 2016, Breton et al., 2011, Cevik and Kilic, 2018, Espinosa-González and Normand, 2019, Gilbert et al., 2015)
**Flexibility & integration with existing systems**	Building on existing models of successes Flexibility in choosing teams and participation in decisions Integration into public health services. Full integration with Health District services and working closely with the local community	(Aggarwal and Williams, 2019, Cevik and Kilic, 2018, Espinosa-González and Normand, 2019, Ghiotto et al., 2018, Goldman et al., 2010)
**Evidence-based performance**	Evidence-based balanced approach Developing a comprehensive medicines policy to include all important areas	(Aggarwal and Williams, 2019, Atun et al., 2016, Goldman et al., 2010, Kanavos et al., 2009)
**Communication and interaction & team working**	Community and provider partnerships Transparency, consultation, and open communication Communication and interaction between the innovators and the adopters Foster collective spaces for reflection, discussion, and practice to occur by stakeholders. Create an enabling environment, to provide flexibility to adopters Strengthening the functional organization and cooperation,	(Aggarwal and Williams, 2019, Atun et al., 2007, Atun et al., 2006, Fausto et al., 2018, Goldman et al., 2010, Lin et al., 2015, Massuda et al., 2018)
**Government/ Political support**	Being a priority of healthcare policymakers and receiving strong support from the government. More effective participation at the state and federal levels Government support with health insurance	(Atun et al., 2006, Carvalho et al., 2014, Ahmedov, 2014, Mendes et al., 2018)
**Empowerment**	Formulation of policies to facilitate and guide the managerial activity and training of managers. Empowering community-level structures Teaching all the teams in one course and enabling the teams to work together. Informing healthcare planners, educators, policymakers, professional bodies, and pharmacists through advocacy and education	(Bheekie and Bradley, 2016, Carvalho et al., 2014, Hailemariam et al., 2018, Mendes et al., 2018)

In the analysis of ‘limitations’ in the studies that have mentioned limitations of PHC reforms, 27 codes were extracted which were classified into 6 categories/themes (economic, management, service delivery, staff, structure, type of services), as shown in Table 9. Requiring substantial investment by the government, lack of an economic evaluation, mainly physician-centric governance, hospital-based system and focusing decisions at the top levels of the organization were the common limitations of many countries in primary health care innovation.

**Table 9.** Limitations of PHC initiatives

Limitations	Instances/examples in studies	References
**Economic**	Lack of an economic evaluation Requiring substantial investment by the government from new funding Sustaining health expenditure and human resources at levels that ensure timely access to high quality Requiring further investment for PHC Community-based services	(Abos Mendizabal et al., 2013, Aggarwal, 2009, Aggarwal and Williams, 2019, Atun et al., 2007, Glazier, 2012, Jaakkimainen et al., 2011, Nickell et al., 2020)
**Management**	Inequities in health status and health behavior Much more additional research is needed Decision-making still mostly centralized at the provincial level Low managerial capacity and accountability at the district level Dual authority for PHC	(Atun et al., 2016, Atun et al., 2007, Bheekie and Bradley, 2016, Rabkin et al., 2015)
**Service delivery**	Mainly physician-centric governance The improper schedule & extended visits Government focus on the delivery of ‘frontline’ medical services	(Aggarwal and Williams, 2019, Booth et al., 2016, Glazier, 2012, Jaakkimainen et al., 2011, Nickell et al., 2020)
**Staff**	Severe shortages of health professionals working in PHC, particularly in rural areas The absence of a gate-keeping function.	(Badora-Musiał et al., 2021, Bhattacharyya et al., 2011)
**Structure**	Hospital-based system Diversity of occupational categories Objective aspects connected to the organization of work. Lack of insurance coverage for community health facilities, Competition between primary and secondary care, Low utilization of facilities in PHC Integration problem between primary care and public health services. Fewer follow-up visits	(Atun et al., 2006, Bhattacharyya et al., 2011, Cevik and Kilic, 2018, Espinosa-González and Normand, 2019, Fausto et al., 2018, Massuda et al., 2018)
**Type of services**	Many challenges in adapting its health system better to serve the needs of its people in an efficient and sustainable fashion Manifestation of previously unmet need Curative or personal health services focused and relatively less attention on public/population health services	(Booth et al., 2016, Lahariya, 2019, Ahmedov, 2014, Tatar et al., 2011)

## Discussion

Reforming and reshaping healthcare is a widespread endeavor in numerous countries, constituting a protracted and challenging process. This trend has gained momentum, particularly since the inception of the current century (Kezunovic et al., 2013). This study endeavors to synthesize the diverse experiences of various nations in the realm of innovations and reforms within their healthcare systems.

To date, compelling evidence underscores the efficacy of PHC in attaining the fundamental objectives of healthcare systems. In recent years, noteworthy strides in reforms and innovations within this type of care, especially in low- and middle-income countries, have accentuated its success, particularly in dimensions such as access and equity (Kruk et al., 2010). Nonetheless, the task of fortifying health systems to enhance the prevailing state of service delivery and implementing cost-effective interventions, with varying approaches across different countries, is inherently intricate. This complexity stems from the influence of financial and human resources, coupled with the distinct political perspectives of each country. Moreover, effective governance, financial and delivery structures within health systems, and adept implementation strategies are imperative (Lewin et al., 2008).

Divergent viewpoints also exist regarding the constituent elements of health systems. An alternative perspective introduces a taxonomy of health system arrangements, which further categorizes and distinguishes between governance arrangements (pertaining to political, economic, and administrative authority in health system management), financial arrangements (encompassing funding and incentive systems, as well as financing), delivery arrangements (including human resources for health and service delivery), and interventions (comprising programs, services, and technologies) (Lavis et al., 2002; Europe, 2006). Notably, many explanations of health system elements overlook the crucial aspect of implementation strategies supporting the utilization of cost-effective interventions (Grol and Grimshaw, 2003).

The health systems of various countries have endeavored to implement changes and reforms across diverse aspects and elements of their PHC, tailoring these initiatives to address specific needs and existing challenges. Each nation has strategically planned innovations and reforms, each with distinct aims and goals. The outcomes of these endeavors have yielded results and consequences, sometimes aligning with their original objectives and at other times deviating from them.

Our findings indicate a discernible trend where countries are increasingly modifying their primary care systems with overarching aims and goals. These include enhancing the quality of care and mitigating the likelihood of hospitalization, establishing an integrated model spanning primary, hospital, and home levels, ensuring superior patient management, facilitating better access to comprehensive health services, fostering an interprofessional care model, restructuring primary care, refining referral systems, enhancing the coordination of care, and transitioning care away from hospitals, among other objectives. Notably, the long-term outcomes of these changes have consistently manifested in improvements in the overall health of the society, increased equity in health, enhanced access to services, improved responsiveness of the health system, and heightened efficiency in service provision. These achievements align with the parameters outlined in the six building block framework.

Concerning the interaction with the government in innovative PHC ‘stewardship/leadership’, various systems exhibit different approaches, categorized as government-dependent, independent, coordinated, or participatory. For instance, Canada’s Family Health Networks (FHNs) exemplify a coordinated system where physicians, either physically co-located or working virtually, emphasize system coordination (Aggarwal and Williams, 2019). In government-dependent systems, tasks such as planning, monitoring, and setting regulations often involve government intervention. In Estonia, for instance, the government takes on responsibilities for planning and regulatory functions (Koppel et al., 2009). Similarly, Bosnia and Herzegovina adopts a decentralized approach, with a federal ministry facilitating services through contracts between insurers and PHC providers (Atun et al., 2006). Brazil showcases decentralization in public health monitoring (de Mello et al., 2017). In China, the government assumes a central role in community health services, where services are delivered by centers under government leadership. This underscores the significant influence of the government in shaping and overseeing PHC initiatives (mention the source if available) (Lin et al., 2015). Our findings indicate a general trend over the past 22 years wherein new PHC structures have transitioned from governmental control to more participatory models involving municipalities. This shift has been substantiated by several studies (Lahariya, 2019, Nickell et al., 2020) which assert that management by municipalities can yield superior outcomes in health management and contribute to the enhancement of health indicators. This is attributed to the comprehensive understanding that local managers possess regarding their regions, enabling them to have a profound knowledge of the health and social challenges faced by the residents in those areas.

In terms of ‘payment’ methods, PHC systems have undergone diverse experiences. Certain countries, like Canada, have consistently expressed a clear preference for the combined payment method, emphasizing its use over the years (Aggarwal and Williams, 2019). A crucial point highlighted in our findings is that Fee-for-Service payments were exclusively in the form of combined payments within PHC systems, with no separate instances of utilization.

Within combined payments, bonus and incentive payments played a significant role, addressing various factors such as enhancing access and providing specialized services. It is noteworthy that some argue that, considering the nature of PHC services, which may not induce additional needs, using the combined payment method is a suitable approach. Nevertheless, regardless of the payment method chosen, models for PHC should ideally be flexible, forward-looking, and oriented towards achieving end outcomes rather than solely focusing on process measures. Such models contribute to both patient and provider satisfaction (Azimzadeh et al., 2023, Bazemore et al., 2018).

In the context of ‘services provided’ within the primary care systems of countries spearheading reforms, there is a notable emphasis on prevention and early patient identification. Consequently, preventive and diagnostic services, including laboratory tests and imaging, are particularly focused on screening for chronic diseases such as diabetes, cardiovascular disease, and cancer. Additionally, acknowledging the contemporary living conditions, mental health has been integrated into the primary care structure in a significant number of countries.

Beyond these aspects, several countries have directed their attention to health promotion services. By offering services such as behavioral health, physical exercise programs, promotion activities addressing obesity and healthy lifestyle behaviors, environmental health services, and communicable disease surveillance; they aim to achieve long-term results and prevent diseases from permeating communities. This approach aligns with the fundamental nature of PHC, which centers on delivering services at the family and community levels, with the goal of reducing the incidence and spread of diseases within communities. (Bheekie and Bradley, 2016, Ditta and Ahmed, 2019, Matulis and Lloyd, 2018). The linkage between the services provided and payment methods is crucial. Shifting payment methods towards health-oriented care with long-term outcomes improves both the quantity and quality of these services. For instance, when payments are directed towards services such as self-care education, healthy lifestyle promotion, and screening, providers prioritize these essential aspects. Therefore, paying careful attention to payment methods is fundamental when planning the types of services to be prioritized.

However, in certain countries, like Canada, the overarching goal has been to encompass all types of services and provide comprehensive care within the structure of the primary care system (Aggarwal, 2009; Aggarwal and Williams, 2019; Goldman et al., 2010).

Concerning the ‘health workforce’, various countries have employed diverse job fields. However, a noteworthy revelation from our findings is the recent trend in numerous countries towards utilizing multi-tasking and multi-skilled personnel. This includes professionals such as general practitioners, family physicians, community health workers, nurses, and clinical staff. This shift has been underscored in studies related to PHC initiatives, emphasizing the efficiency and cost-effectiveness of such personnel. Moreover, this approach presents opportunities for expanding coverage and mitigating human resource shortages. Studies also highlight the pivotal role of working conditions, personnel training, and collaborative activities within health teams in enhancing the productivity of human resources. These aspects have been consistently emphasized in the literature. (Rule et al., 2014; Lewin et al., 2008; Rabkin et al., 2015).

The significance of collecting and utilizing ‘information’ in the health sector has been underscored in various studies, playing a pivotal role in the PHC initiatives of different countries. For instance, Spain’s PHC innovations are grounded in information use, emphasizing continuous improvement and real innovation through relevant software utilization and the creation of extensive databases. In Spain, the implementation of a variety of care programs based on the health and social needs of patients was sought through the utilization of variables in electronic Health Information Systems (Abos Mendizabal et al., 2013; Doñate-Martínez, 2017, Prades and Borràs, 2011). Similarly, numerous countries have pursued innovations in PHC by seeking electronic prescriptions, utilizing electronic health records, maintaining computer-based medicine records, and facilitating information exchange between clinical settings to enhance efficiency and reduce paperwork and duplication (Atun et al., 2016, de Mello et al., 2017, Lahariya, 2019). Technological advances have paved the way for online health services like remote monitoring and consultation, enabling greater service accessibility irrespective of geographic location and empowering patients to actively participate in their clinical treatment. This active role encompasses patient participation, involvement, adherence, and compliance (Menichetti et al., 2016). Digital tools such as patient portals and mobile applications offer convenience to both providers and patients, providing easier access to health records and clinical expertise while minimizing the collection of health data. These tools have the potential to enhance patient experiences and transform the patient-provider relationship from a paternalistic to a patient-centered model (Perakslis and Ginsburg, 2021, Ravoire et al., 2017, Schofield et al., 2019).

Despite the evident benefits, countries encountered challenges and limitations in implementing reforms and initiatives within their existing PHC. To address these challenges, they employed necessary considerations and policies, aiming to eliminate or reduce the adverse effects caused by the implementation of new systems and ensure compatibility. Various challenges – economic, structural, and managerial – sometimes emerge unexpectedly in the path of PHC innovations and initiatives. However, drawing from the experiences of others and the findings of similar studies, it is possible to mitigate and reduce their effects. For example, Abos Mendizabal et al.’s study in 2013 emphasizes the need for a comprehensive economic evaluation of the value of ideas against the cost of their development and implementation before initiating innovations and reforms (Abos Mendizabal et al., 2013). Rabkin et al.’s study in 2015 suggests that more research may be necessary to determine optimal approaches to program design and delivery, emphasizing that changes should not be rushed (Rabkin et al., 2015). Fausto MCR et al.’s study in 2018 points out that some challenges are related to the objective aspects of work organization, advocating for operational and pilot investigations before the final launch (Fausto et al., 2018).

## Conclusion

In conclusion, it can be affirmed that the fortification of PHC necessitates the commitment, coordination, and collaboration of a broad spectrum of organizations, both health-related and unrelated, requiring a comprehensive health-oriented approach within the country’s macro-policies.

The findings of this study offer distinct insights for policymakers and health system managers, potentially yielding valuable outcomes. These outcomes include advocating for the prioritization of primary and community-oriented care over secondary and patient-oriented care, expanding and enhancing existing information systems in PHC beyond primary data collection, reinforcing health teams and health service packages, as well as cultivating trust, participation, and engagement among the public, involving them in the management of their health. Considering the profound and undeniable consequences of these measures across various dimensions, their necessity and importance are increasingly apparent to all stakeholders.
